# Bayesian estimates of linkage disequilibrium

**DOI:** 10.1186/1471-2156-8-36

**Published:** 2007-06-25

**Authors:** Paola Sebastiani, María M Abad-Grau

**Affiliations:** 1 Department of Biostatistics, Boston University School of Public Health, Boston, MA 02118, USA; 2 Software Engineering Department, University of Granada, Granada 18071, Spain

## Abstract

**Background:**

The maximum likelihood estimator of *D' *– a standard measure of linkage disequilibrium – is biased toward disequilibrium, and the bias is particularly evident in small samples and rare haplotypes.

**Results:**

This paper proposes a Bayesian estimation of *D' *to address this problem. The reduction of the bias is achieved by using a prior distribution on the pair-wise associations between single nucleotide polymorphisms (SNP)s that increases the likelihood of equilibrium with increasing physical distances between pairs of SNPs. We show how to compute the Bayesian estimate using a stochastic estimation based on MCMC methods, and also propose a numerical approximation to the Bayesian estimates that can be used to estimate patterns of LD in large datasets of SNPs.

**Conclusion:**

Our Bayesian estimator of *D' *corrects the bias toward disequilibrium that affects the maximum likelihood estimator. A consequence of this feature is a more objective view about the extent of linkage disequilibrium in the human genome, and a more realistic number of tagging SNPs to fully exploit the power of genome wide association studies.

## Background

Single nucleotide polymorphisms (SNPs) are an invaluable resource to identify regions of the human genome that may be associated with disease. A key to this process is *linkage disequilibrium *(LD) that is defined as the non-random association between the alleles of SNPs [1]. Although LD may occur between SNPs that are not in linkage but are associated, we will focus on the LD due to the spatial structure of the genome. In this situation, the non-random association implies that pairs of alleles in the same haplotype occur differently from what we would expect in a random pairing and several measures of LD have been proposed to capture the departure from independent pairing of the alleles of SNPs [2].

In this paper we will limit attention to *D*, its normalized version *D'*, and the well known bias of the Maximum Likelihood Estimate (MLE) of *D' *toward disequilibrium [2,3]. This bias is particularly large in small samples and SNPs with rare alleles to the point that SNPs whose alleles occur independently may be inferred to be in strong LD [4]. However, relying on small samples to identify patterns of LD is not unusual: for example, the *International HapMap Project *(IHMP) aims to establish genome-wide patterns of LD using genotype data of at most 30 trios or 45 unrelated individuals [5]. The genotype data typed in this small number of samples are used to describe the extent of LD in the human genome, and derive a map of the haplotypes and the SNPs that are sufficient to tag the human genome. These results will have a deep impact on genome wide association studies and, in particular, inferring larger blocks of LD than the real ones may lead to the selection of an insufficient number of SNPs and hence decrease the power of genome wide association studies. In this scenario, biasing the estimate of LD toward equilibrium appears to be a safer alternative.

Several solutions have been proposed to reduce the bias of the MLE of *D' *toward disequilibrium [4]. A pragmatic solution is to impose some "ad hoc" threshold on the minimum allele frequency (MAF) of those SNPs that can be used to infer the pattern of LD [6]. Imposing this threshold leads to a non-random selection of SNPs and may introduce ascertainment bias [7,8]. The thought behind our approach is that the bias of the MLEs of *D *and *D' *is due to the lack of information in the data to discriminate between equilibrium and different magnitude of disequilibrium, and any attempt to correct this bias is due to fail as it was acknowledged in [3]. However, it is known that, on average, the strength of LD due to linkage decreases as the physical distance between SNPs increases [9,10]. Therefore, we propose a Bayesian estimator of *D' *that allows us to integrate data with prior information about the pattern of LD decay. To this end, we use a prior distribution on the pairwise dependencies between different SNPs that is a decreasing function of their physical distance. We show how to compute the posterior estimate of *D' *using Markov Chain Monte Carlo methods, and provide a numerical approximation that can be used for fast estimation of LD in large regions of the human genome. As we show in simulated and real data from the IHMP, the effect of the prior distribution is to drastically reduce the bias toward disequilibrium even in small samples, and to remove the need of arbitrary thresholds on the MAF. We also show that, compared to the MLE, our estimators lead to infer patterns of LD decay that are much closer to published results [10], and confirms the existence of haplotype blocks as regions of low recombination. The method is implemented in a computer program called *Bayesian Linkage *(*BLink*) [11].

## Results and discussion

### The traditional *D *and *D'*

Given two SNPs *L*_1 _and *L*_2_, with alleles *A*/*a *and *B*/*b*, *A *and *B *the major alleles, we define the probability of the haplotype *ij *by *p*_*ij *_= _*p*_(*L*_1 _= *i*, *L*_2 _= *j*), *i *= *A, a*, *j *= *B, b*. As in [12], we assume the relation *p*_*A *_≥ *p*_*B *_on the probabilities *p*_*A *_= *p*(*L*_1 _= *A*), *p*_*B *_= *p*(*L*_2 _= *B*) from which the inequality *p*_*Ab *_≥ *p*_*aB *_follows.

The two SNPs are in *linkage equilibrium *when the co-occurrence of two alleles on the same haplotype is random, e.g. *p*_*ij *_= *p*_*i*_*p*_*j *_for all *i *= *A, a*, *j *= *B, b*. On the other hand, LD implies some form of dependency in the alleles on the same haplotype and hence departure from independence of the probabilities *p*_*ij*_. Although there are many ways to measure departure from independence in a 2 × 2 table [13], a widely used measure of LD is the parameter *D *defined by

*D *= *p*_*AB *_- *p*_*A*_*p*_*B *_- *p*_*a*_*p*_*b *_≤ *D *≤ *p*_*a*_*p*_*B*_.

Because the domain of *D *is a function of the allele frequencies, different normalization methods have been proposed to facilitate the interpretation [2]. The most common one is the measure D′s
 MathType@MTEF@5@5@+=feaafiart1ev1aaatCvAUfKttLearuWrP9MDH5MBPbIqV92AaeXatLxBI9gBaebbnrfifHhDYfgasaacH8akY=wiFfYdH8Gipec8Eeeu0xXdbba9frFj0=OqFfea0dXdd9vqai=hGuQ8kuc9pgc9s8qqaq=dirpe0xb9q8qiLsFr0=vr0=vr0dc8meaabaqaciaacaGaaeqabaqabeGadaaakeaacuWGebargaqbamaaBaaaleaacqWGZbWCaeqaaaaa@2F64@ that was suggested by Lewontin [14] and is defined as *D*/max *D*:

D′s={pAB−pApBpapbifD≤0pAB−pApBpapBifD>0
 MathType@MTEF@5@5@+=feaafiart1ev1aaatCvAUfKttLearuWrP9MDH5MBPbIqV92AaeXatLxBI9gBaebbnrfifHhDYfgasaacH8akY=wiFfYdH8Gipec8Eeeu0xXdbba9frFj0=OqFfea0dXdd9vqai=hGuQ8kuc9pgc9s8qqaq=dirpe0xb9q8qiLsFr0=vr0=vr0dc8meaabaqaciaacaGaaeqabaqabeGadaaakeaacuWGebargaqbamaaBaaaleaacqWGZbWCaeqaaOGaeyypa0ZaaiqabeaafaqaaeGadaaabaWaaSaaaeaacqWGWbaCdaWgaaWcbaGaemyqaeKaemOqaieabeaakiabgkHiTiabdchaWnaaBaaaleaacqWGbbqqaeqaaOGaemiCaa3aaSbaaSqaaiabdkeacbqabaaakeaacqWGWbaCdaWgaaWcbaGaemyyaegabeaakiabdchaWnaaBaaaleaacqWGIbGyaeqaaaaaaOqaaiabbMgaPjabbAgaMbqaaiabdseaejabgsMiJkabicdaWaqaamaalaaabaGaemiCaa3aaSbaaSqaaiabdgeabjabdkeacbqabaGccqGHsislcqWGWbaCdaWgaaWcbaGaemyqaeeabeaakiabdchaWnaaBaaaleaacqWGcbGqaeqaaaGcbaGaemiCaa3aaSbaaSqaaiabdggaHbqabaGccqWGWbaCdaWgaaWcbaGaemOqaieabeaaaaaakeaacqqGPbqAcqqGMbGzaeaacqWGebarcqGH+aGpcqaIWaamaaaacaGL7baaaaa@5D34@

D′s
 MathType@MTEF@5@5@+=feaafiart1ev1aaatCvAUfKttLearuWrP9MDH5MBPbIqV92AaeXatLxBI9gBaebbnrfifHhDYfgasaacH8akY=wiFfYdH8Gipec8Eeeu0xXdbba9frFj0=OqFfea0dXdd9vqai=hGuQ8kuc9pgc9s8qqaq=dirpe0xb9q8qiLsFr0=vr0=vr0dc8meaabaqaciaacaGaaeqabaqabeGadaaakeaacuWGebargaqbamaaBaaaleaacqWGZbWCaeqaaaaa@2F64@ is defined in the interval [-1, 1], with D′s
 MathType@MTEF@5@5@+=feaafiart1ev1aaatCvAUfKttLearuWrP9MDH5MBPbIqV92AaeXatLxBI9gBaebbnrfifHhDYfgasaacH8akY=wiFfYdH8Gipec8Eeeu0xXdbba9frFj0=OqFfea0dXdd9vqai=hGuQ8kuc9pgc9s8qqaq=dirpe0xb9q8qiLsFr0=vr0=vr0dc8meaabaqaciaacaGaaeqabaqabeGadaaakeaacuWGebargaqbamaaBaaaleaacqWGZbWCaeqaaaaa@2F64@ = ± 1 describing perfect disequilibrium and D′s
 MathType@MTEF@5@5@+=feaafiart1ev1aaatCvAUfKttLearuWrP9MDH5MBPbIqV92AaeXatLxBI9gBaebbnrfifHhDYfgasaacH8akY=wiFfYdH8Gipec8Eeeu0xXdbba9frFj0=OqFfea0dXdd9vqai=hGuQ8kuc9pgc9s8qqaq=dirpe0xb9q8qiLsFr0=vr0=vr0dc8meaabaqaciaacaGaaeqabaqabeGadaaakeaacuWGebargaqbamaaBaaaleaacqWGZbWCaeqaaaaa@2F64@ = 0 describing equilibrium. It is also common to take the absolute value of D′s
 MathType@MTEF@5@5@+=feaafiart1ev1aaatCvAUfKttLearuWrP9MDH5MBPbIqV92AaeXatLxBI9gBaebbnrfifHhDYfgasaacH8akY=wiFfYdH8Gipec8Eeeu0xXdbba9frFj0=OqFfea0dXdd9vqai=hGuQ8kuc9pgc9s8qqaq=dirpe0xb9q8qiLsFr0=vr0=vr0dc8meaabaqaciaacaGaaeqabaqabeGadaaakeaacuWGebargaqbamaaBaaaleaacqWGZbWCaeqaaaaa@2F64@, say *D' *= |D′s
 MathType@MTEF@5@5@+=feaafiart1ev1aaatCvAUfKttLearuWrP9MDH5MBPbIqV92AaeXatLxBI9gBaebbnrfifHhDYfgasaacH8akY=wiFfYdH8Gipec8Eeeu0xXdbba9frFj0=OqFfea0dXdd9vqai=hGuQ8kuc9pgc9s8qqaq=dirpe0xb9q8qiLsFr0=vr0=vr0dc8meaabaqaciaacaGaaeqabaqabeGadaaakeaacuWGebargaqbamaaBaaaleaacqWGZbWCaeqaaaaa@2F64@| to have a measure in the interval [0,1]. This is for example the default measure of LD in the popular program HaploView [6].

### Maximum likelihood estimation

Suppose now that we have a data set of *N *individuals and *n *= 2*N *known haplotypes for the two SNPs (we assume here known phase for all haplotypes and discuss the phasing issue at the end of this section). We denote by *n*_*ij *_(*i *= *A, a*, *j *= *B, b*) the frequencies of the four haplotypes, and by *n*_*i *_and *n*_*j *_the allele frequencies with *n*_*A *_≥ *n*_*B*_. Assuming that the four haplotypes follow a multinomial distribution with probabilities *p*_*ij*_, the likelihood function can be written as:

l(pij)∝∏ijpijnij
 MathType@MTEF@5@5@+=feaafiart1ev1aaatCvAUfKttLearuWrP9MDH5MBPbIqV92AaeXatLxBI9gBaebbnrfifHhDYfgasaacH8akY=wiFfYdH8Gipec8Eeeu0xXdbba9frFj0=OqFfea0dXdd9vqai=hGuQ8kuc9pgc9s8qqaq=dirpe0xb9q8qiLsFr0=vr0=vr0dc8meaabaqaciaacaGaaeqabaqabeGadaaakeaacqWGSbaBcqGGOaakcqWGWbaCdaWgaaWcbaGaemyAaKMaemOAaOgabeaakiabcMcaPiabg2Hi1oaarafabaGaemiCaa3aa0baaSqaaiabdMgaPjabdQgaQbqaaiabd6gaUnaaBaaameaacqWGPbqAcqWGQbGAaeqaaaaaaSqaaiabdMgaPjabdQgaQbqab0Gaey4dIunaaaa@42F8@

and the MLE of *p*_*ij*_, *p*_*A *_and *p*_*B *_are

p^ij=nABn;p^A=nAn;p^B=nBn
 MathType@MTEF@5@5@+=feaafiart1ev1aaatCvAUfKttLearuWrP9MDH5MBPbIqV92AaeXatLxBI9gBaebbnrfifHhDYfgasaacH8akY=wiFfYdH8Gipec8Eeeu0xXdbba9frFj0=OqFfea0dXdd9vqai=hGuQ8kuc9pgc9s8qqaq=dirpe0xb9q8qiLsFr0=vr0=vr0dc8meaabaqaciaacaGaaeqabaqabeGadaaakeaafaqabeqadaaabaGafmiCaaNbaKaadaWgaaWcbaGaemyAaKMaemOAaOgabeaakiabg2da9maalaaabaGaemOBa42aaSbaaSqaaiabdgeabjabdkeacbqabaaakeaacqWGUbGBaaGaei4oaSdabaGafmiCaaNbaKaadaWgaaWcbaGaemyqaeeabeaakiabg2da9maalaaabaGaemOBa42aaSbaaSqaaiabdgeabbqabaaakeaacqWGUbGBaaGaei4oaSdabaGafmiCaaNbaKaadaWgaaWcbaGaemOqaieabeaakiabg2da9maalaaabaGaemOBa42aaSbaaSqaaiabdkeacbqabaaakeaacqWGUbGBaaaaaaaa@4906@

from which we derive the MLE of *D*, D′s
 MathType@MTEF@5@5@+=feaafiart1ev1aaatCvAUfKttLearuWrP9MDH5MBPbIqV92AaeXatLxBI9gBaebbnrfifHhDYfgasaacH8akY=wiFfYdH8Gipec8Eeeu0xXdbba9frFj0=OqFfea0dXdd9vqai=hGuQ8kuc9pgc9s8qqaq=dirpe0xb9q8qiLsFr0=vr0=vr0dc8meaabaqaciaacaGaaeqabaqabeGadaaakeaacuWGebargaqbamaaBaaaleaacqWGZbWCaeqaaaaa@2F64@ and *D'*:

D^=p^AB−p^Ap^B
 MathType@MTEF@5@5@+=feaafiart1ev1aaatCvAUfKttLearuWrP9MDH5MBPbIqV92AaeXatLxBI9gBaebbnrfifHhDYfgasaacH8akY=wiFfYdH8Gipec8Eeeu0xXdbba9frFj0=OqFfea0dXdd9vqai=hGuQ8kuc9pgc9s8qqaq=dirpe0xb9q8qiLsFr0=vr0=vr0dc8meaabaqaciaacaGaaeqabaqabeGadaaakeaacuWGebargaqcaiabg2da9iqbdchaWzaajaWaaSbaaSqaaiabdgeabjabdkeacbqabaGccqGHsislcuWGWbaCgaqcamaaBaaaleaacqWGbbqqaeqaaOGafmiCaaNbaKaadaWgaaWcbaGaemOqaieabeaaaaa@38F3@

D^′s=D^p^ap^BI(D^≥0)+D^p^ap^bI(D^<0)
 MathType@MTEF@5@5@+=feaafiart1ev1aaatCvAUfKttLearuWrP9MDH5MBPbIqV92AaeXatLxBI9gBaebbnrfifHhDYfgasaacH8akY=wiFfYdH8Gipec8Eeeu0xXdbba9frFj0=OqFfea0dXdd9vqai=hGuQ8kuc9pgc9s8qqaq=dirpe0xb9q8qiLsFr0=vr0=vr0dc8meaabaqaciaacaGaaeqabaqabeGadaaakeaacuWGebargaqcgaqbamaaBaaaleaacqWGZbWCaeqaaOGaeyypa0ZaaSaaaeaacuWGebargaqcaaqaaiqbdchaWzaajaWaaSbaaSqaaiabdggaHbqabaGccuWGWbaCgaqcamaaBaaaleaacqWGcbGqaeqaaaaakiabdMeajjabcIcaOiqbdseaezaajaGaeyyzImRaeGimaaJaeiykaKIaey4kaSYaaSaaaeaacuWGebargaqcaaqaaiqbdchaWzaajaWaaSbaaSqaaiabdggaHbqabaGccuWGWbaCgaqcamaaBaaaleaacqWGIbGyaeqaaaaakiabdMeajjabcIcaOiqbdseaezaajaGaeyipaWJaeGimaaJaeiykaKcaaa@4BF5@

D′=|D^′s|=−D^p^ap^BI(D^≥0)+D^p^ap^bI(D^<0)
 MathType@MTEF@5@5@+=feaafiart1ev1aaatCvAUfKttLearuWrP9MDH5MBPbIqV92AaeXatLxBI9gBaebbnrfifHhDYfgasaacH8akY=wiFfYdH8Gipec8Eeeu0xXdbba9frFj0=OqFfea0dXdd9vqai=hGuQ8kuc9pgc9s8qqaq=dirpe0xb9q8qiLsFr0=vr0=vr0dc8meaabaqaciaacaGaaeqabaqabeGadaaakeaacuWGebargaqbaiabg2da9maaemaabaGafmiraqKbaKGbauaadaWgaaWcbaGaem4CamhabeaaaOGaay5bSlaawIa7aiabg2da9iabgkHiTmaalaaabaGafmiraqKbaKaaaeaacuWGWbaCgaqcamaaBaaaleaacqWGHbqyaeqaaOGafmiCaaNbaKaadaWgaaWcbaGaemOqaieabeaaaaGccqWGjbqscqGGOaakcuWGebargaqcaiabgwMiZkabicdaWiabcMcaPiabgUcaRmaalaaabaGafmiraqKbaKaaaeaacuWGWbaCgaqcamaaBaaaleaacqWGHbqyaeqaaOGafmiCaaNbaKaadaWgaaWcbaGaemOyaigabeaaaaGccqWGjbqscqGGOaakcuWGebargaqcaiabgYda8iabicdaWiabcMcaPaaa@5227@

where *I*(*x *∈ *X*) is the indicator function defined as *I*(*x *∈ *X*) = 1 if *x *∈ *X *and 0 otherwise. Note that:

• D^=−p^ap^b
 MathType@MTEF@5@5@+=feaafiart1ev1aaatCvAUfKttLearuWrP9MDH5MBPbIqV92AaeXatLxBI9gBaebbnrfifHhDYfgasaacH8akY=wiFfYdH8Gipec8Eeeu0xXdbba9frFj0=OqFfea0dXdd9vqai=hGuQ8kuc9pgc9s8qqaq=dirpe0xb9q8qiLsFr0=vr0=vr0dc8meaabaqaciaacaGaaeqabaqabeGadaaakeaacuWGebargaqcaiabg2da9iabgkHiTiqbdchaWzaajaWaaSbaaSqaaiabdggaHbqabaGccuWGWbaCgaqcamaaBaaaleaacqWGIbGyaeqaaaaa@35AC@ whenever *n*_*ab *_= 0, so that D^′s
 MathType@MTEF@5@5@+=feaafiart1ev1aaatCvAUfKttLearuWrP9MDH5MBPbIqV92AaeXatLxBI9gBaebbnrfifHhDYfgasaacH8akY=wiFfYdH8Gipec8Eeeu0xXdbba9frFj0=OqFfea0dXdd9vqai=hGuQ8kuc9pgc9s8qqaq=dirpe0xb9q8qiLsFr0=vr0=vr0dc8meaabaqaciaacaGaaeqabaqabeGadaaakeaacuWGebargaqcgaqbamaaBaaaleaacqWGZbWCaeqaaaaa@2F73@ = -1 and D^′
 MathType@MTEF@5@5@+=feaafiart1ev1aaatCvAUfKttLearuWrP9MDH5MBPbIqV92AaeXatLxBI9gBaebbnrfifHhDYfgasaacH8akY=wiFfYdH8Gipec8Eeeu0xXdbba9frFj0=OqFfea0dXdd9vqai=hGuQ8kuc9pgc9s8qqaq=dirpe0xb9q8qiLsFr0=vr0=vr0dc8meaabaqaciaacaGaaeqabaqabeGadaaakeaacuWGebargaqcgaqbaaaa@2DD8@ = 1;

• D^=p^ap^B
 MathType@MTEF@5@5@+=feaafiart1ev1aaatCvAUfKttLearuWrP9MDH5MBPbIqV92AaeXatLxBI9gBaebbnrfifHhDYfgasaacH8akY=wiFfYdH8Gipec8Eeeu0xXdbba9frFj0=OqFfea0dXdd9vqai=hGuQ8kuc9pgc9s8qqaq=dirpe0xb9q8qiLsFr0=vr0=vr0dc8meaabaqaciaacaGaaeqabaqabeGadaaakeaacuWGebargaqcaiabg2da9iqbdchaWzaajaWaaSbaaSqaaiabdggaHbqabaGccuWGWbaCgaqcamaaBaaaleaacqWGcbGqaeqaaaaa@347F@ whenever *n*_*aB *_= 0, so that D^′s
 MathType@MTEF@5@5@+=feaafiart1ev1aaatCvAUfKttLearuWrP9MDH5MBPbIqV92AaeXatLxBI9gBaebbnrfifHhDYfgasaacH8akY=wiFfYdH8Gipec8Eeeu0xXdbba9frFj0=OqFfea0dXdd9vqai=hGuQ8kuc9pgc9s8qqaq=dirpe0xb9q8qiLsFr0=vr0=vr0dc8meaabaqaciaacaGaaeqabaqabeGadaaakeaacuWGebargaqcgaqbamaaBaaaleaacqWGZbWCaeqaaaaa@2F73@ = 1 and D^′
 MathType@MTEF@5@5@+=feaafiart1ev1aaatCvAUfKttLearuWrP9MDH5MBPbIqV92AaeXatLxBI9gBaebbnrfifHhDYfgasaacH8akY=wiFfYdH8Gipec8Eeeu0xXdbba9frFj0=OqFfea0dXdd9vqai=hGuQ8kuc9pgc9s8qqaq=dirpe0xb9q8qiLsFr0=vr0=vr0dc8meaabaqaciaacaGaaeqabaqabeGadaaakeaacuWGebargaqcgaqbaaaa@2DD8@ = 1.

These two facts determine the bias toward disequilibrium of D′s
 MathType@MTEF@5@5@+=feaafiart1ev1aaatCvAUfKttLearuWrP9MDH5MBPbIqV92AaeXatLxBI9gBaebbnrfifHhDYfgasaacH8akY=wiFfYdH8Gipec8Eeeu0xXdbba9frFj0=OqFfea0dXdd9vqai=hGuQ8kuc9pgc9s8qqaq=dirpe0xb9q8qiLsFr0=vr0=vr0dc8meaabaqaciaacaGaaeqabaqabeGadaaakeaacuWGebargaqbamaaBaaaleaacqWGZbWCaeqaaaaa@2F64@ and *D' *that can lead to infer that two SNPs are in disequilibrium when they are actually in equilibrium. For example, the expected number of haplotypes *ab *in a sample of *n *haplotypes between two SNPs in equilibrium is *np*_*a*_*p*_*b*_. If both *p*_*a *_and *p*_*b *_are smaller than 0.1, then the minimum sample size *n *that yields an expected number of haplotypes *ab *greater than 1 is 100, and this number goes up to 400 when both *p*_*a *_and *p*_*b *_are 0.05. Therefore, data from small samples and rare alleles do not provide information to discriminate between equilibrium and disequilibrium, while the MLE of *D' *returns its maximum value consistent with perfect disequilibrium. This situation is well known and supported by simulation studies. Teare and coauthors [4] showed the extent of this bias through extensive simulations in which they examined the effect of sample size, MAF and strength of LD on the MLE of *D'*. Their study suggested that the bias is severe in small samples (less than 100 subjects), when both alleles are rare (MAF less than 0.05), and the two SNPs are in equilibrium. An "ad hoc" solution consists of disregarding those SNPs with a MAF below some threshold. Although this reduces the bias of the MLEs, it introduces an ascertainment bias that may impact the inferred pattern of LD [7].

### Bayesian approach

Our Bayesian estimator is based on the following intuition: on average, the magnitude of disequilibrium between two SNPs decreases at exponential rate with their physical distance. We use this information to build a conjugate prior distribution on the parameters *p*_*ij *_with the property that, a priori, the larger the distance between two SNPs, the more likely the two SNPs are in linkage equilibrium. The standard conjugate prior to a multinomial distribution is a Dirichlet distribution with density function defined as:

p(pij)∝∏ijpijαij−1αij>0.
 MathType@MTEF@5@5@+=feaafiart1ev1aaatCvAUfKttLearuWrP9MDH5MBPbIqV92AaeXatLxBI9gBaebbnrfifHhDYfgasaacH8akY=wiFfYdH8Gipec8Eeeu0xXdbba9frFj0=OqFfea0dXdd9vqai=hGuQ8kuc9pgc9s8qqaq=dirpe0xb9q8qiLsFr0=vr0=vr0dc8meaabaqaciaacaGaaeqabaqabeGadaaakeaafaqabeqacaaabaGaemiCaaNaeiikaGIaemiCaa3aaSbaaSqaaiabdMgaPjabdQgaQbqabaGccqGGPaqkcqGHDisTdaqeqbqaaiabdchaWnaaDaaaleaacqWGPbqAcqWGQbGAaeaaiiGacqWFXoqydaWgaaadbaGaemyAaKMaemOAaOgabeaaliabgkHiTiabigdaXaaaaeaacqWGPbqAcqWGQbGAaeqaniabg+GivdaakeaacqWFXoqydaWgaaWcbaGaemyAaKMaemOAaOgabeaakiabg6da+iabicdaWiabc6caUaaaaaa@4C97@

Given data *n*_*ij*_, the posterior distribution is still a Dirichlet distribution with density function:

p(pij)∝∏ijpijαij+nij−1
 MathType@MTEF@5@5@+=feaafiart1ev1aaatCvAUfKttLearuWrP9MDH5MBPbIqV92AaeXatLxBI9gBaebbnrfifHhDYfgasaacH8akY=wiFfYdH8Gipec8Eeeu0xXdbba9frFj0=OqFfea0dXdd9vqai=hGuQ8kuc9pgc9s8qqaq=dirpe0xb9q8qiLsFr0=vr0=vr0dc8meaabaqaciaacaGaaeqabaqabeGadaaakeaacqWGWbaCcqGGOaakcqWGWbaCdaWgaaWcbaGaemyAaKMaemOAaOgabeaakiabcMcaPiabg2Hi1oaarafabaGaemiCaa3aa0baaSqaaiabdMgaPjabdQgaQbqaaGGaciab=f7aHnaaBaaameaacqWGPbqAcqWGQbGAaeqaaSGaey4kaSIaemOBa42aaSbaaWqaaiabdMgaPjabdQgaQbqabaWccqGHsislcqaIXaqmaaaabaGaemyAaKMaemOAaOgabeqdcqGHpis1aaaa@4A55@

in which the prior hyper-parameters *α*_*ij *_are updated into *α*_*ij *_+ *n*_*ij*_. The prior means of the parameters *p*_*ij *_are *α*_*ij*_/*α*_*T*_, where *α*_*T *_= ∑_*ij*_*α*_*ij*_. The posterior means become *E*(*p*_*ij*_|*n*) = (*α*_*ij *_+ *n*_*ij*_)/(*α*_*T *_+ *n*) and can be used as point estimates of the parameters. Furthermore, the posterior distributions of the marginal probabilities *p*_*A *_and *p*_*B *_follow Beta distributions with hyper-parameters (*α*_*A *_+ *n*_*A*_, *α*_*a *_+ *n*_*a*_) and (*α*_*B *_+ *n*_*B*_, *α*_*b *_+ *n*_*b*_), for *α*_*A *_= *α*_*AB *_+ *α*_*Ab*_, *α*_*a *_= *α*_*T *_- *α*_*A*_, *α*_*B *_= *α*_*AB *_+ *α*_*aB*_, and *α*_*b *_= *α*_*T *_- *α*_*B*_.

The inference on the parameters *D*, *D*_*s *_and *D' *is more complex. First, we note that we can write these parameters as follows:

*D *= *p*_*AB *_- *p*_*A*_*p*_*B*_

Ds=Dpapbp(D<0)+DpapBp(D≥0)
 MathType@MTEF@5@5@+=feaafiart1ev1aaatCvAUfKttLearuWrP9MDH5MBPbIqV92AaeXatLxBI9gBaebbnrfifHhDYfgasaacH8akY=wiFfYdH8Gipec8Eeeu0xXdbba9frFj0=OqFfea0dXdd9vqai=hGuQ8kuc9pgc9s8qqaq=dirpe0xb9q8qiLsFr0=vr0=vr0dc8meaabaqaciaacaGaaeqabaqabeGadaaakeaacqWGebardaWgaaWcbaGaem4Camhabeaakiabg2da9maalaaabaGaemiraqeabaGaemiCaa3aaSbaaSqaaiabdggaHbqabaGccqWGWbaCdaWgaaWcbaGaemOyaigabeaaaaGccqWGWbaCcqGGOaakcqWGebarcqGH8aapcqaIWaamcqGGPaqkcqGHRaWkdaWcaaqaaiabdseaebqaaiabdchaWnaaBaaaleaacqWGHbqyaeqaaOGaemiCaa3aaSbaaSqaaiabdkeacbqabaaaaOGaemiCaaNaeiikaGIaemiraqKaeyyzImRaeGimaaJaeiykaKcaaa@4BF6@

D′=−Dpapbp(D<0)+DpapBp(D≥0)
 MathType@MTEF@5@5@+=feaafiart1ev1aaatCvAUfKttLearuWrP9MDH5MBPbIqV92AaeXatLxBI9gBaebbnrfifHhDYfgasaacH8akY=wiFfYdH8Gipec8Eeeu0xXdbba9frFj0=OqFfea0dXdd9vqai=hGuQ8kuc9pgc9s8qqaq=dirpe0xb9q8qiLsFr0=vr0=vr0dc8meaabaqaciaacaGaaeqabaqabeGadaaakeaacuWGebargaqbaiabg2da9iabgkHiTmaalaaabaGaemiraqeabaGaemiCaa3aaSbaaSqaaiabdggaHbqabaGccqWGWbaCdaWgaaWcbaGaemOyaigabeaaaaGccqWGWbaCcqGGOaakcqWGebarcqGH8aapcqaIWaamcqGGPaqkcqGHRaWkdaWcaaqaaiabdseaebqaaiabdchaWnaaBaaaleaacqWGHbqyaeqaaOGaemiCaa3aaSbaaSqaaiabdkeacbqabaaaaOGaemiCaaNaeiikaGIaemiraqKaeyyzImRaeGimaaJaeiykaKcaaa@4B4A@

**Figure 1:**
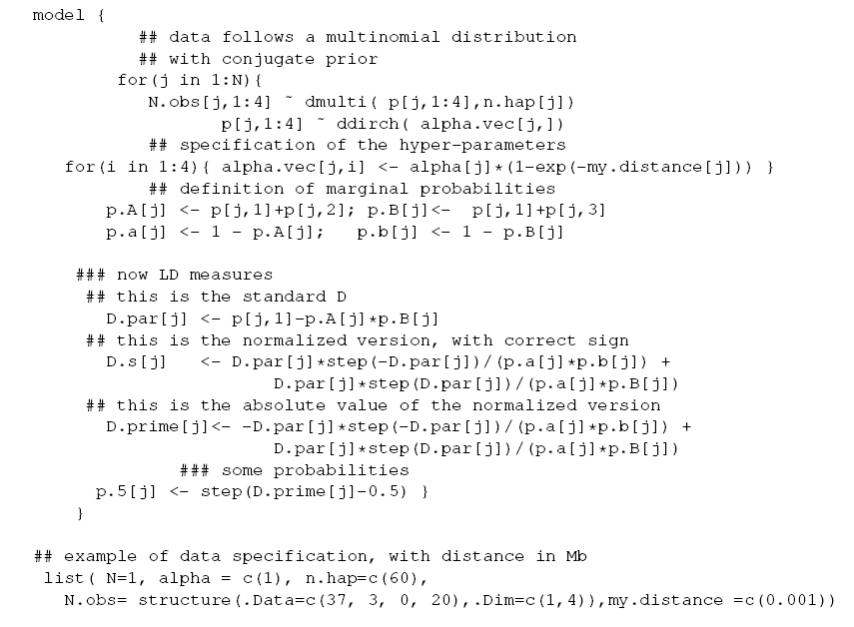
Model Specification in WinBugs 1.4.

Equations (9) and (10) define the parameters *D*_*s *_and *D' *as mixtures of two components, with weights *p*(*D *< 0) and *p*(*D *≥ 0). The two components are non linear functions of the parameters *p*_*ij*_, as is the parameter *D*, and make the exact inference on these parameters intractable. However, we can resort on Markov Chain Monte Carlo methods to generate a sample of values of either parameters from their posterior distribution that can be used for further inference. In Figure 1, we provide a model description that can be used in Winbugs 1.4 to generate samples from the posterior distributions of the parameters *D*, *D*_*s *_and *D'*.

#### Choice of the prior distribution

To complete the specification of the Bayesian model, we need to provide values for the hyper-parameters. Because we wish to encode the information that departure from equilibrium of any two SNPs is a function of their physical distance, we define:

*α*_*ij *_= *α*(1 - exp(-*d*))

with *α *> 0, and the parameter *d *that represents the physical distance between the two SNPs in Mb (1 Mb = 1, 000 nucleotide bases). With this choice of hyper-parameters, the prior means of the probabilities *p*_*ij *_are *E*(*p*_*ij*_) = 1/4 for all *i *= *A, a*, *j *= *B, b*, so that, a priori, the two SNPs are expected to be in equilibrium. On the other hand, the posterior means of *p*_*ij *_can be written as:

E(pij|n)=nn+αTnijn+αTn+αTαijαT=nn+4α(1−exp⁡(−d))nijn+4α(1−exp⁡(−d))n+4α(1−exp⁡(−d))14
 MathType@MTEF@5@5@+=feaafiart1ev1aaatCvAUfKttLearuWrP9MDH5MBPbIqV92AaeXatLxBI9gBaebbnrfifHhDYfgasaacH8akY=wiFfYdH8Gipec8Eeeu0xXdbba9frFj0=OqFfea0dXdd9vqai=hGuQ8kuc9pgc9s8qqaq=dirpe0xb9q8qiLsFr0=vr0=vr0dc8meaabaqaciaacaGaaeqabaqabeGadaaakeaafaqaaeGadaaabaGaemyrauKaeiikaGIaemiCaa3aaSbaaSqaaiabdMgaPjabdQgaQbqabaGccqGG8baFcqWGUbGBcqGGPaqkaeaacqGH9aqpaeaadaWcaaqaaiabd6gaUbqaaiabd6gaUjabgUcaRGGaciab=f7aHnaaBaaaleaacqWGubavaeqaaaaakmaalaaabaGaemOBa42aaSbaaSqaaiabdMgaPjabdQgaQbqabaaakeaacqWGUbGBaaGaey4kaSYaaSaaaeaacqWFXoqydaWgaaWcbaGaemivaqfabeaaaOqaaiabd6gaUjabgUcaRiab=f7aHnaaBaaaleaacqWGubavaeqaaaaakmaalaaabaGae8xSde2aaSbaaSqaaiabdMgaPjabdQgaQbqabaaakeaacqWFXoqydaWgaaWcbaGaemivaqfabeaaaaaakeaaaeaacqGH9aqpaeaadaWcaaqaaiabd6gaUbqaaiabd6gaUjabgUcaRiabisda0iab=f7aHjabcIcaOiabigdaXiabgkHiTiGbcwgaLjabcIha4jabcchaWjabcIcaOiabgkHiTiabdsgaKjabcMcaPiabcMcaPaaadaWcaaqaaiabd6gaUnaaBaaaleaacqWGPbqAcqWGQbGAaeqaaaGcbaGaemOBa4gaaiabgUcaRmaalaaabaGaeGinaqJae8xSdeMaeiikaGIaeGymaeJaeyOeI0IagiyzauMaeiiEaGNaeiiCaaNaeiikaGIaeyOeI0IaemizaqMaeiykaKIaeiykaKcabaGaemOBa4Maey4kaSIaeGinaqJae8xSdeMaeiikaGIaeGymaeJaeyOeI0IagiyzauMaeiiEaGNaeiiCaaNaeiikaGIaeyOeI0IaemizaqMaeiykaKIaeiykaKcaamaalaaabaGaeGymaedabaGaeGinaqdaaaaaaaa@8FAC@

and, hence, as a weighted average of *n*_*ij*_/*n *(the MLE estimates of *p*_*ij*_) and the prior probabilities 1/4. The first weight *n*/(*n *+ 4*α*(1 - exp(-*d*))) is an increasing function of the sample size *n*, and a decreasing function of *α *and *d*, while the second weight 4*α*(1 - exp(-*d*))/(*n *+ 4*α*(1 - exp(-*d*))) is an increasing function of *α *and *d*, and a decreasing function of *n*. Therefore, for large sample sizes, the posterior means of *p*_*ij *_approach the MLEs. This is consistent with the fact that, in large samples, the effect of the prior distribution on the posterior distribution becomes negligible. However, when the distance *d *decreases, the function 1 - exp(-*d*) approaches 0, and the weight *n*/(*n *+ 4*α*(1 - exp(-*d*))) approaches 1, so that the Bayesian estimate becomes closer to the MLE. In the limiting case *d *= 0, or *α *= 0, the two estimates are identical. For fixed *α *and increasing distance (essentially *d *> 0.5*Mb*), the second weight approaches its maximum value 4*α*/(*n *+ 4*α*), and larger values of *α *further increase the weight given to the prior mean. To contain the effect of the prior distribution, we use *α *= 1 and simulation studies that are described in the next section show that this choice produces a good trade-off between robustness and bias.

**Figure 2:**
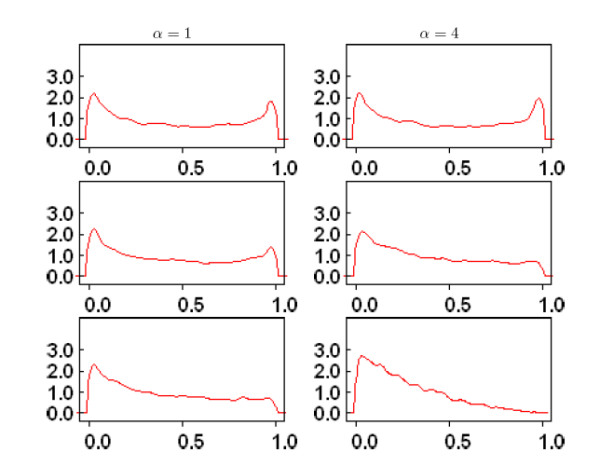
Example of prior distribution of the parameter *D' *for *α *= 1 (column 1) and *α *= 4 (column 2) and increasing distance between the two SNPs: row 1 *d *= 0.001 Mb; row 2 *d *= 0.1 Mb and row 3 *d *= 0.5 Mb. The x-axis displays *D' *and the y-axes displays the empirical estimate of the density function inferred with the program WinBugs 1.4.

In the absence of a closed form expression for the prior distributions of the parameters *D*, D′s
 MathType@MTEF@5@5@+=feaafiart1ev1aaatCvAUfKttLearuWrP9MDH5MBPbIqV92AaeXatLxBI9gBaebbnrfifHhDYfgasaacH8akY=wiFfYdH8Gipec8Eeeu0xXdbba9frFj0=OqFfea0dXdd9vqai=hGuQ8kuc9pgc9s8qqaq=dirpe0xb9q8qiLsFr0=vr0=vr0dc8meaabaqaciaacaGaaeqabaqabeGadaaakeaacuWGebargaqbamaaBaaaleaacqWGZbWCaeqaaaaa@2F64@ and *D'*, we investigated the effect of *α *and *d *by generating stochastic estimates of their prior densities. Figure 2 shows the prior density of *D'*generated by Markov Chain Monte Carlo simulations in Winbugs 1.4 for *α *= 1, 4 and distance ranging between *d *= 0.001 *Mb *and *d *= 0.5 *Mb*. The estimates are based on a sample of 5, 000 iterations, after an initial burns in of 1, 000 iterations. The two plots in the top panel depict the prior density of *D' *for *α *= 1 (left panel) and *α *= 4 (right panel) when the distance between the two SNPs is *d *= 0.001 *Mb*. The prior density peaks at the extreme value *D' *= 0 that represents equilibrium between the two SNPs, and *D' *= 1 that represents perfect disequilibrium. Therefore, for small distance, this bimodal distribution makes these opposite situations almost equally likely. The effect of larger *α *is to shift the density toward equilibrium: for example the probability that *D' *< 0.5 is 0.54 when *α *= 1 and becomes 0.57 when *α *= 4. The effect of increasing *α *appears negligible in this situation, but it is more evident when the two SNPs are at a larger distance. For example, the two plots in the second panel depict the prior density of *D' *when *d *= 0.1 *Mb*. Compared to the densities in the top panel, now the prior densities are slightly skewed toward 0, thus making disequilibrium less likely. Once again, greater values of *α *(right panel) increase the skewness toward equilibrium. The two plots in the bottom panel show the prior densities when the distance between the two SNPs is 0.5 *Mb *and confirm the increasing weight given to equilibrium for larger distance *d *and larger *α *values: the prior density now assigns probability 0.68 to the event *D' *< 0.5, when *α *= 1, and probability 0.84 to the same event when *α *= 4. It is interesting to observe that, as the distance between SNPs decreases, then the prior hyperparameters approach 0, and the Bayesian estimates approach the MLE estimates. Accordingly, the prior distribution moves mass from *D' *= 0 to *D' *= 1 as the distance decreases and this explains the bimodal shape of the prior density in the top two panels.

**Figure 3:**
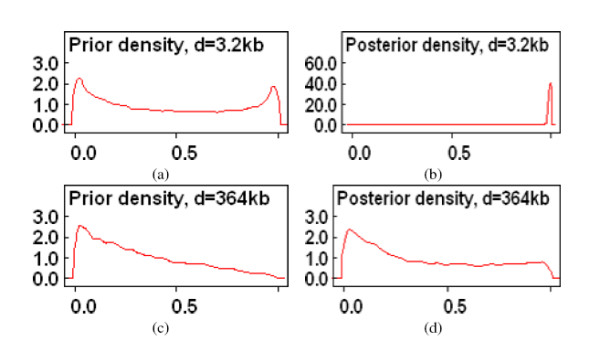
Examples of prior to posterior analysis using the data in Table 2. The x-axes display *D' *and the y-axes display the empirical estimate of the density function inferred with the program WinBugs 1.4. Figure (a) is the prior density of *D' *to measure the LD between the SNPs S1 and S2 that are at a distance of 3.2 kb. Figure (b) displays the posterior density, given the data in Table 1, and shows that data overwhelm the prior distribution when the distance is relatively small. Figure (c) is the prior density of *D' *to measure the LD between the SNPs S1 and S3 that are now more distant (364 kb). Figure (d) shows the posterior distribution, given the same data in Table 1, and shows now the relatively smaller effect of the data on the prior density.

As an example, Table 1 displays the frequencies of the four haplotypes *AB*, *Ab*, *aB *and *ab *that were observed between SNPs S1 and S2 Chromosome 22, at the positions 15040669, 15043944. These are real data that were derived from the thirty trios of the CEPH population (Utah residents with ancestry from northern and western Europe) who provided the DNA samples for the IHMP [5]. The observed haplotype frequencies are consistent with the hypothesis of linkage equilibrium, because the expected number of haplotypes *ab *is 0.5 under equilibrium and the assumption that the population allele frequencies equal the marginal estimates *p*_*a *_= 0.03 and *p*_*b *_= 0.14. However, the lack of observed haplotypes *ab *could be due to perfect LD between each pair of SNPs. Given that the physical distance between S1 and S2 is 0.0032 Mb, and the average *D' *in chromosome 22 ranges between 0.8 and 1 for SNPs that are within 0.01 Mb, and becomes less than 0.5 for SNPs that are distant more than 0.1 Mb [5], it is likely that S1 and S2 are in disequilibrium. Consider now a third SNP S3 in the position 15405264 of chromosome 22. The frequencies of the four haplotypes between S1 and S3 is the same as in Table 1 but now the physical distance between these two SNPs is 0.364 Mb. Given the extent of LD, equilibrium is more likely between S1 and S3, although the haplotype frequencies are the same. The MLE of *D' *is 1 in both cases, with the same confidence interval, while the Bayesian estimate of *D' *changes with the distance between the two SNPs. The plots in Figure 3 show the prior distribution of *D' *(plots on the left) and the posterior distribution (plots on the right) between the closest SNPs (first row) and the first and third SNP (second row). A priori, disequilibrium and equilibrium are equally likely when the two SNPs are very close, but the posterior distribution is dominated by the data and the left skewness is consistent with the hypothesis of disequilibrium between the two SNPs. The point estimate of *D' *is 0.96 with 95% credible interval (0.37, 1). The effect of the same data on the distribution of *D' *for SNPs that are further apart is however more contained: the posterior distribution of *D' *remains skewed to the right, the point estimates of *D' *is 0.39, and the 95% credible interval is (0, 0.97) showing the large uncertainty.

**Table 1:** Data are derived from the 30 trios of the CEPH population. Haplotype frequencies between two SNPs S1 and S2 in chromosome 22.

SNP	SNP	S1	
S2	*B*	*b*	
*A*	99	17	116
*a*	4	0	4

	103	17	120

#### Approximate estimates

In practical applications, we have computed the Bayesian estimate of *D' *for regions with at most 200 SNPs that would correspond to examining a block of approximately 500 kb assuming one SNP every 2.5 kb. However, if the focus is generating a point estimate of the parameters to be able to display LD over large regions or an entire chromosome as we have shown in [15], resort to MCMC methods may become unfeasible. It is possible to compute the exact posterior mean of *D*, and from this we can derive approximate estimates of *D*_*s *_and *D' *based on a Taylor expansion. To this end, we replace the weights *p*(*D *< 0) and *p*(*D *≥ 0) in Equations (9) and (10) by the indicator functions *I*(*E*(*D*|*n*) < 0) and *I*(*E*(*D*|*n*) ≥ 0), and the expectation of the non linear functions *D*/max *D *by the first order Taylor expansion:

E(D|n)=E(pAB|n)−E(pA|n)E(pB|n)=αAB+nABαT+n−(αA+nA)(αB+nB)(αT+n)2E(D′s|n)≈E(D|n){(αT+n)2(αa+na)(αb+nb)I(E(D|n)<0)+(αT+n)(αa+na)I(E(D|n)≥0)}E(D′|n)≈E(D|n){−(αT+n)2(αa+na)(αb+nb)I(E(D|n)<0)+(αT+n)(αa+na)I(E(D|n)≥0)}
 MathType@MTEF@5@5@+=feaafiart1ev1aaatCvAUfKttLearuWrP9MDH5MBPbIqV92AaeXatLxBI9gBaebbnrfifHhDYfgasaacH8akY=wiFfYdH8Gipec8Eeeu0xXdbba9frFj0=OqFfea0dXdd9vqai=hGuQ8kuc9pgc9s8qqaq=dirpe0xb9q8qiLsFr0=vr0=vr0dc8meaabaqaciaacaGaaeqabaqabeGadaaakeaafaqaaeWadaaabaGaemyrauKaeiikaGIaemiraqKaeiiFaWNaemOBa4MaeiykaKcabaGaeyypa0dabaGaemyrauKaeiikaGIaemiCaa3aaSbaaSqaaiabdgeabjabdkeacbqabaGccqGG8baFcqWGUbGBcqGGPaqkcqGHsislcqWGfbqrcqGGOaakcqWGWbaCdaWgaaWcbaGaemyqaeeabeaakiabcYha8jabd6gaUjabcMcaPiabdweafjabcIcaOiabdchaWnaaBaaaleaacqWGcbGqaeqaaOGaeiiFaWNaemOBa4MaeiykaKIaeyypa0ZaaSaaaeaaiiGacqWFXoqydaWgaaWcbaGaemyqaeKaemOqaieabeaakiabgUcaRiabd6gaUnaaBaaaleaacqWGbbqqcqWGcbGqaeqaaaGcbaGae8xSde2aaSbaaSqaaiabdsfaubqabaGccqGHRaWkcqWGUbGBaaGaeyOeI0YaaSaaaeaacqGGOaakcqWFXoqydaWgaaWcbaGaemyqaeeabeaakiabgUcaRiabd6gaUnaaBaaaleaacqWGbbqqaeqaaOGaeiykaKIaeiikaGIae8xSde2aaSbaaSqaaiabdkeacbqabaGccqGHRaWkcqWGUbGBdaWgaaWcbaGaemOqaieabeaakiabcMcaPaqaaiabcIcaOiab=f7aHnaaBaaaleaacqWGubavaeqaaOGaey4kaSIaemOBa4MaeiykaKYaaWbaaSqabeaacqaIYaGmaaaaaaGcbaGaemyrauKaeiikaGIafmiraqKbauaadaWgaaWcbaGaem4CamhabeaakiabcYha8jabd6gaUjabcMcaPaqaaiabgIKi7cqaaiabdweafjabcIcaOiabdseaejabcYha8jabd6gaUjabcMcaPmaacmqabaWaaSaaaeaacqGGOaakcqWFXoqydaWgaaWcbaGaemivaqfabeaakiabgUcaRiabd6gaUjabcMcaPmaaCaaaleqabaGaeGOmaidaaaGcbaGaeiikaGIae8xSde2aaSbaaSqaaiabdggaHbqabaGccqGHRaWkcqWGUbGBdaWgaaWcbaGaemyyaegabeaakiabcMcaPiabcIcaOiab=f7aHnaaBaaaleaacqWGIbGyaeqaaOGaey4kaSIaemOBa42aaSbaaSqaaiabdkgaIbqabaGccqGGPaqkaaGaemysaKKaeiikaGIaemyrauKaeiikaGIaemiraqKaeiiFaWNaemOBa4MaeiykaKIaeyipaWJaeGimaaJaeiykaKIaey4kaSYaaSaaaeaacqGGOaakcqWFXoqydaWgaaWcbaGaemivaqfabeaakiabgUcaRiabd6gaUjabcMcaPaqaaiabcIcaOiab=f7aHnaaBaaaleaacqWGHbqyaeqaaOGaey4kaSIaemOBa42aaSbaaSqaaiabdggaHbqabaGccqGGPaqkaaGaemysaKKaeiikaGIaemyrauKaeiikaGIaemiraqKaeiiFaWNaemOBa4MaeiykaKIaeyyzImRaeGimaaJaeiykaKcacaGL7bGaayzFaaaabaGaemyrauKaeiikaGIafmiraqKbauaacqGG8baFcqWGUbGBcqGGPaqkaeaacqGHijYUaeaacqWGfbqrcqGGOaakcqWGebarcqGG8baFcqWGUbGBcqGGPaqkdaGadeqaaiabgkHiTmaalaaabaGaeiikaGIae8xSde2aaSbaaSqaaiabdsfaubqabaGccqGHRaWkcqWGUbGBcqGGPaqkdaahaaWcbeqaaiabikdaYaaaaOqaaiabcIcaOiab=f7aHnaaBaaaleaacqWGHbqyaeqaaOGaey4kaSIaemOBa42aaSbaaSqaaiabdggaHbqabaGccqGGPaqkcqGGOaakcqWFXoqydaWgaaWcbaGaemOyaigabeaakiabgUcaRiabd6gaUnaaBaaaleaacqWGIbGyaeqaaOGaeiykaKcaaiabdMeajjabcIcaOiabdweafjabcIcaOiabdseaejabcYha8jabd6gaUjabcMcaPiabgYda8iabicdaWiabcMcaPiabgUcaRmaalaaabaGaeiikaGIae8xSde2aaSbaaSqaaiabdsfaubqabaGccqGHRaWkcqWGUbGBcqGGPaqkaeaacqGGOaakcqWFXoqydaWgaaWcbaGaemyyaegabeaakiabgUcaRiabd6gaUnaaBaaaleaacqWGHbqyaeqaaOGaeiykaKcaaiabdMeajjabcIcaOiabdweafjabcIcaOiabdseaejabcYha8jabd6gaUjabcMcaPiabgwMiZkabicdaWiabcMcaPaGaay5Eaiaaw2haaaaaaaa@203C@

The main source of error in this approximation is due to replacing the probability *P*(*D *≥ 0) by the indicator function. When we are in a clear situation of disequilibrium, the probability of the event (*D *< 0) is almost 0 or 1, and the approximate posterior expectation of *D*_*s *_and *D' *approaches the exact values. When *p*(*D *< 0) is far from 0 and 1, then the error increases and biases the estimates toward disequilibrium. This is consistent with the fact that the approximation is close to the MLE and therefore suffers of some bias toward disequilibrium. However, we will show with results of simulations in the next section that this bias is smaller. Because of this similarity with the MLE, we will refer to these approximate estimates as the maximum a posteriori (MAP).

#### Unknown phase

When the genotype data are unphased, the ML estimation uses the EM algorithm to infer the unknown phase given the distribution of known haplotypes [16]. We adopt the same procedure for the calculation of the MAP estimates. Given the frequencies of known haplotypes, *n*_*ij*_, *i *= *A, a, j *= *B, b*, the algorithm first computes the MAP estimates of the haplotype frequencies *p*_*ij*_, and then alternates an expectation step to replace the unphased haplotypes by their expected phase and a maximization step to compute the MAP estimates using observed and expected haplotypes. The algorithm typically converges in less than 4 steps. Unknown haplotypes are regarded as missing values in the stochastic analysis, so that they become parameters of the model and are estimated within the Gibbs sampling algorithm. We also note that, when the genotype data are from trios, we use all phased haplotypes to compute the initial frequencies, regardless of whether they are transmitted from parents to offspring.

The method is implemented in the computer program *BLink *that is developed in C++ and is available from the supplementary web site [11]. The software accepts genotype data from either unrelated individuals or nuclear families consisting of two parents and one child.

### Evaluation

We examined the performance of the Bayesian estimator in three groups of simulated data and a real data set derived from the IHMP. All data used in this evaluation are available from the supplementary web site [11].

## Materials and methods

### Group 1

The objectives of the first simulation study were (1) to compare the performance of the Bayesian estimates and the MLE for different sample sizes and small values of the MAF, and (2) to assess the accuracy of the MAP approximation to the stochastic estimates of *D*_*s *_and *D'*. We generated samples of 60, 120, 240 haplotypes, in which we modeled the true *D' *as *D' *= exp(-*d*) for a distance *d *ranging from 0 to 0.5 Mb. For each value of *D' *and each sample size, we generated 1,000 samples of haplotypes by using the joint probability of haplotypes defined by Equation (1), with *p*_*B *_generated from a uniform distribution in the interval [0.5; 0.9) and *p*_*A *_generated from a uniform distribution in the interval [*p*_*B*_; 0.95). In each simulated sample, we computed the MLE, and the MAP estimate of *D*_*s*_, as well as the stochastic estimate of *D*_*s *_using Gibbs sampling. To compute the stochastic estimates we run the chain for an initial burn-in of 1, 000 iterations and then based the inference on a second sample of 1, 000 iterations. We used as point estimate the median value of the simulated sample and *α *= 1 in each analysis.

### Group 2

In this second set, we generated a sample of 1, 000 individuals in a region of 0.5 Mb with the program MS that simulates genotype data under a variety of neutral models [17]. We considered a population of 1 million individuals, a mutation rate of 10*E *- 9 per base pair, and a recombination rate of 8 × 10*E *- 9 between adjacent base pairs per generation. Only 10% of the 8080 SNPs in the sample of 1, 000 individuals were randomly selected and, from this sample, we randomly generated subsamples of sizes 60, 120, 240 and 480 haplotypes. In the absence of "true" values for *D'*, we studied the decay of LD inferred by the MLE and the MAP estimator for increasing physical distances, versus the LD decay inferred in the original sample of 1, 000 individuals. Each point in the plot is the average estimate of *D' *for all the SNPs within a physical distance of *d *± 0.01 Mb. By averaging the LD between pairs of SNPs at increasing distance, these plots are used to summarize the decay of LD over large regions [18,10]. Ascertainment bias was assessed by repeating the analysis with these thresholds on the MAF: 0, 0.05, 0.1, 0.2. Sensitivity to the prior distribution was assessed by repeating the analysis for *α *= 0.25, 1, 2, 4.

### Group 3

To examine the robustness of the MAP estimators, we also generated data under a different model of allele frequency, linkage disequilibrium and population differentiation that is implemented in the software COSI [19]. We simulated a sample of 1, 000 individuals under the calibrated model for the European population that considers bottlenecks, migration and recombination hotspots spacing 0.085 Mb [19]. We randomly selected 10% of the generated 32452 SNPs and from this sample we randomly selected subsamples of 60, 120, 240 and 480 haplotypes. We produced LD decay plots using the thresholds 0, 0.05, 0.1, 0.2 on the MAF and the range of *α *values that were used for the analysis of the simulated data in group 2.

### Real data

Real data were obtained from the first phase of the IHMP [5]. We used genotype data of the 30 trios of the CEPH and Yoruba in Ibadan, Nigeria and chromosome 22 because its pattern of LD has been widely studied [9]. This chromosome was genotyped in 19120 and 19854 SNPs in the CEPH and Yoruba samples. We produced LD decay plots using the thresholds on the MAF and the range of *α *values that we used for the analysis of the simulated data in group 2. We also produced more informative graphical displays of pairwise LD, by generating bi-dimensional maps similar to those generated by the program Haploview, but with a lower resolution to enable the display of LD over larger regions. The maps were generated with the program BMapBuilder [15] using the MLE and the MAP estimate of *D'*.

## Results

**Figure 4:**
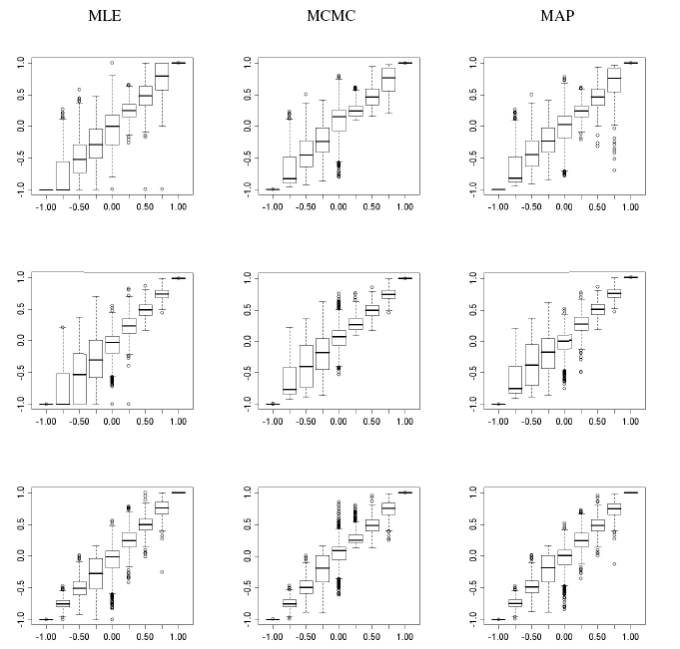
Distribution of the estimates of D′s
 MathType@MTEF@5@5@+=feaafiart1ev1aaatCvAUfKttLearuWrP9MDH5MBPbIqV92AaeXatLxBI9gBaebbnrfifHhDYfgasaacH8akY=wiFfYdH8Gipec8Eeeu0xXdbba9frFj0=OqFfea0dXdd9vqai=hGuQ8kuc9pgc9s8qqaq=dirpe0xb9q8qiLsFr0=vr0=vr0dc8meaabaqaciaacaGaaeqabaqabeGadaaakeaacuWGebargaqbamaaBaaaleaacqWGZbWCaeqaaaaa@2F64@ (y-axis) versus the true *D' *(x-axis) computed using ML (column 1), MCMC (column 2) and the MAP approximation (column 3), for 3 different sample sizes: *n *= 60 (row 1); *n *= 120 (row 2); and *n *= 240 (row 3). Each boxplot reports the distribution of the 1,000 estimates generated for each sample size and value of D′s
 MathType@MTEF@5@5@+=feaafiart1ev1aaatCvAUfKttLearuWrP9MDH5MBPbIqV92AaeXatLxBI9gBaebbnrfifHhDYfgasaacH8akY=wiFfYdH8Gipec8Eeeu0xXdbba9frFj0=OqFfea0dXdd9vqai=hGuQ8kuc9pgc9s8qqaq=dirpe0xb9q8qiLsFr0=vr0=vr0dc8meaabaqaciaacaGaaeqabaqabeGadaaakeaacuWGebargaqbamaaBaaaleaacqWGZbWCaeqaaaaa@2F64@.

Figure 4 reports the distribution of the ML, MCMC and MAP estimates of D′s
 MathType@MTEF@5@5@+=feaafiart1ev1aaatCvAUfKttLearuWrP9MDH5MBPbIqV92AaeXatLxBI9gBaebbnrfifHhDYfgasaacH8akY=wiFfYdH8Gipec8Eeeu0xXdbba9frFj0=OqFfea0dXdd9vqai=hGuQ8kuc9pgc9s8qqaq=dirpe0xb9q8qiLsFr0=vr0=vr0dc8meaabaqaciaacaGaaeqabaqabeGadaaakeaacuWGebargaqbamaaBaaaleaacqWGZbWCaeqaaaaa@2F64@ in the data simulated in Group 1, for different values of D′s
 MathType@MTEF@5@5@+=feaafiart1ev1aaatCvAUfKttLearuWrP9MDH5MBPbIqV92AaeXatLxBI9gBaebbnrfifHhDYfgasaacH8akY=wiFfYdH8Gipec8Eeeu0xXdbba9frFj0=OqFfea0dXdd9vqai=hGuQ8kuc9pgc9s8qqaq=dirpe0xb9q8qiLsFr0=vr0=vr0dc8meaabaqaciaacaGaaeqabaqabeGadaaakeaacuWGebargaqbamaaBaaaleaacqWGZbWCaeqaaaaa@2F64@ and sample sizes. The plots on the left show the bias of the MLE of D′s
 MathType@MTEF@5@5@+=feaafiart1ev1aaatCvAUfKttLearuWrP9MDH5MBPbIqV92AaeXatLxBI9gBaebbnrfifHhDYfgasaacH8akY=wiFfYdH8Gipec8Eeeu0xXdbba9frFj0=OqFfea0dXdd9vqai=hGuQ8kuc9pgc9s8qqaq=dirpe0xb9q8qiLsFr0=vr0=vr0dc8meaabaqaciaacaGaaeqabaqabeGadaaakeaacuWGebargaqbamaaBaaaleaacqWGZbWCaeqaaaaa@2F64@ toward disequilibrium that is more apparent for negative values of D′s
 MathType@MTEF@5@5@+=feaafiart1ev1aaatCvAUfKttLearuWrP9MDH5MBPbIqV92AaeXatLxBI9gBaebbnrfifHhDYfgasaacH8akY=wiFfYdH8Gipec8Eeeu0xXdbba9frFj0=OqFfea0dXdd9vqai=hGuQ8kuc9pgc9s8qqaq=dirpe0xb9q8qiLsFr0=vr0=vr0dc8meaabaqaciaacaGaaeqabaqabeGadaaakeaacuWGebargaqbamaaBaaaleaacqWGZbWCaeqaaaaa@2F64@ and samples with less than 120 haplotypes. The median ML estimates of *D' *is -1 when the real *D' *is -0.75 for samples with less than 120 haplotypes, while the median MCMC estimate in 1000 simulations is -.82 in samples with 60 haplotypes and -0.76 in samples with 120 haplotypes. Furthermore, even with sample sizes of less than 60 haplotypes, the MCMC estimates and the MAP approximations are virtually undistinguishable thus suggesting that the screening of LD in large regions can be based on the MAP approximation.

**Figure 5:**
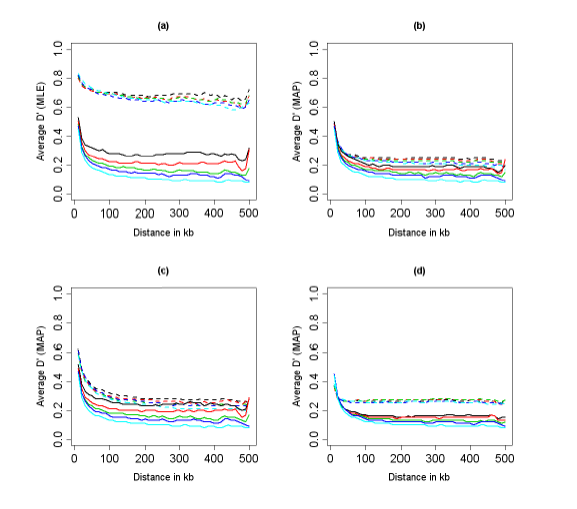
LD decay plots for the data generated in Group 2, based on the MLE (panel a) and the MAP approximation (panel b: *α *= 1, panel c: *α *= 0.25, and panel d: *α *= 4) of *D'*. The line type represents two thresholds on the MAF: 0.0 (dashed line), and 0.05 (continuous line). The line color represents five sample sizes: *n *= 60 (black); *n *= 120 (red); *n *= 240 (green); *n *= 480 (dark blue); *n *= 1000 (pale blue).

**Figure 6:**
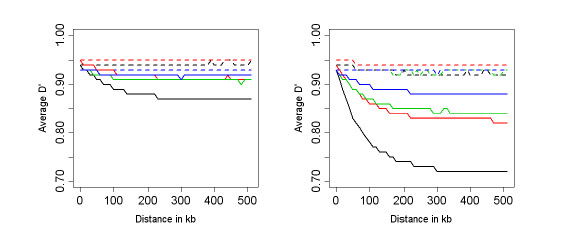
LD decay plots for the data generated in Group 3, with high LD, MAF> 0.05, and sporadic hotspots. The left panel displays the LD decay inferred by using the MLE (dashed lines) and the MAP approximation (continuous line), with *α *= 1. The line color represents four sample sizes: *n *= 60 (black); *n *= 120 (red); *n *= 240 (green); *n *≥ 480 (dark blue). The right panel displays the LD decay for the same data based on the MAP approximation of *D' *with *α *= 0.25 (dashed lines) and *α *= 16 (continuous lines). The line color represents the sample size as in the left panel.

**Figure 7:**
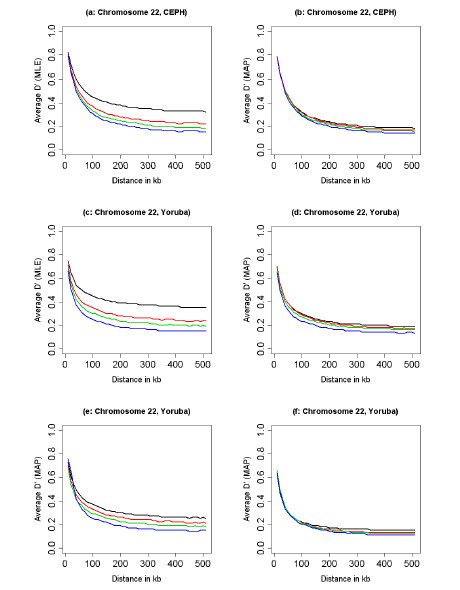
Panels (a) and (b): LD decay plot of chromosome 22, based on the MLE of *D' *(panel a) and the MAP estimator of *D' *(panel b) in the CEPH population. The line color represents four thresholds on the MAF: 0.0 (black); 0.05 (red); 0.10 (green); 0.20 (dark blue). Panels (c) and (d): LD decay plot of chromosome 22, based on the MLE of *D' *(panel c) and the MAP estimator of *D' *(panel d) in the Yoruba population. We used *α *= 1. Panels (e) and (f): LD decay of chromosome 22 in the Yoruba population based on the MAP estimate of *D' *with *α *= 0.25 (panel e) and *α *= 4 (panel f). The line color represents the four thresholds on the MAF as in the other plots.

Figures 5, 6 and 7 show the summary of the LD decay generated with the ML and MAP estimators of *D'*. In each figure, the *x*-axis reports the distance between pairs of SNPs in kb. For each *x *value, the *y*-axis reports the estimate of the average *D' *for all SNPs within a distance *x *± 0.01 Mb. Figure 5 plots the LD decay of the data simulated with the program MS. Panels (a) and (b) display the LD decay generated using the ML and MAP estimates of *D'*, with *α *= 1, and MAF > 0 (dashed lines), MAF > 0.05 (continuous lines), and samples sizes ranging from 60 to 1, 000 represented by different colors. Both the ML and MAP estimates show approximately the same values in the sample of 1, 000 individuals and MAF> 0.05 (continuous lines, pale blue), so that we can consider those values as representative of population values. The LD decay plots based on the MLE of *D' *appear to be extremely sensitive to the threshold chosen for the MAF. For example when the MAF > 0 (dashed lines), the ML estimator leads us to infer long range disequilibrium. In contrast, the MAP estimator produces more consistent results across different thresholds on the MAF, and sample sizes (see also supplementary results in [11]), to the point that we do not need to impose any threshold on the MAF. The plots in panels (c) and (d) display the LD decay generated with the MAP estimator using *α *= 0.25 (c) and *α *= 4 (d). The choice of the prior hyperparameter appears to be influential when all the SNPs are used to estimate the LD decay, with smaller values of *α *that make the MAP estimator more sensitive to the sample size, and larger values of *α *that increase the bias toward equilibrium. These results would suggest choosing *α *= 1 in practical applications, to achieve a good balance between robustness and bias reduction.

The LD decay plots in Figure 6 were generated from the data simulated with the program COSI with long range disequilibrium. The plot on the left displays the average LD computed with the MLE (dashed lines), and the MAP estimator with *α *= 1 (continuous lines), MAF> 0.05, and increasing sample sizes. The bias of the MLE of *D' *now works in favor of this estimator that is able to reproduce the larger values of *D' *compared to the MAP estimator. Although of smaller magnitude, the MAP estimate of *D'*remains consistent with long range disequilibrium. The plot in panel (b) shows the LD decay inferred with the MAP estimator using *α *= 0.25 (dashed line) and *α *= 4 (continuous line). The evident departure from the true pattern of LD when *α *= 4 again suggests choosing a small *α *value, say *α *= 1, to limit the bias toward equilibrium.

Figure 7 plots the LD decay generated with the ML and MAP estimators using the CEPH and Yoruba samples of the IHMP, different thresholds on the MAF, and different *α *values. The results confirm the bias toward disequilibrium of the MLE of *D' *– panels (a) and (c)- when all the SNPs are used in the analysis. The MAP estimator leads to more consistent results for a range of thresholds on the MAF, see panels (b) and (d). These results suggest that, even with a small sample size, we do not need to select SNPs based on the MAF, thus removing the issue of the ascertainment bias. Panels (e) and (f) display the LD decay in Chromosome 22, Yoruba samples, for *α *= 0.25 (e) and *α *= 4 (f). Values of *α *greater than 1 appear to bias the estimator toward equilibrium, while values of *α *smaller than 1 lead to a loss of robustness. Consistently with the analysis of simulated data, the choice *α *= 1 appears to achieve a good balance between robustness and bias reduction.

**Figure 8:**
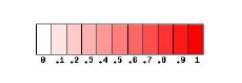
Colors used in bitmaps for different values of *D'*.

**Figure 9:**
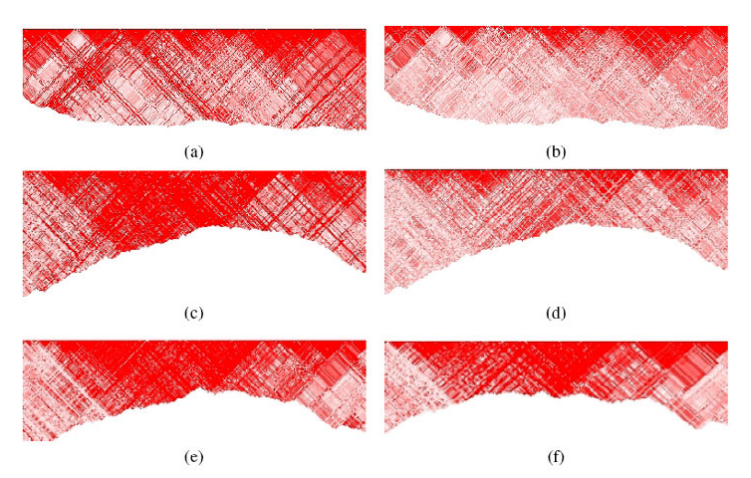
Maps of LD in a region of chromosome 22 based on the MLE of *D' *in the CEPH population (a) and the Yoruba population (c) and the MAP estimator of *D' *in the CEPH population (b) and the Yoruba population (d). The maps in panels (e) and (f) were inferred using the MLE of *D' *for the same chromosome region of the Yoruba population using SNPs with the MAF>0.05 and MAF>0.1. The color coding is described in Figure 8.

Figure 9 provides a two-dimensional display of pairwise LD. Each plot represents the value of *D' *between a pair of SNPs as a colored pixel whose intensity is related to the value of *D'*, see the legend in Figure 8. Higher resolution maps (16 pixels for a pair of SNPs) are available in the supplementary web site. The first row of Figure 9 shows maps of LD for a region of chromosome 22 in the CEPH populations and the second row shows maps of LD for the Yoruba population, using the MLE (first column) and the MAP estimator (second column). These four maps were generated without imposing any threshold on the MAF, while the maps in the third row display the pairwise LD in the same segment of chromosome 22 of the Yoruba population, based on the MLE of *D'*, MAF> 0.05 (panel e) and MAF>0.1 (panel f). The MAP estimator of *D' *reduces the long range LD – maps (b) and (d)- without the need of imposing a threshold on the MAF. This smoothing induced by the MAP estimator has the effect of highlighting blocks of high LD more clearly, compared to the MLE. For example, two blocks are clearly visible on the left of the maps in panel (d) while they are hidden in a larger region of high LD in the map created with the MLE of *D' *(panel c) and even with tighter thresholds on the MAF those two blocks are hardly recognizable (panels e and f). These results provide evidence of a block structure of the human genome that does not appear to be an artifact of low SNP density [5]. However, they also suggest the presence of smaller blocks of LD that may impact on the minimum number of tag SNPs needed to have powerful genome wide association studies.

## Conclusion

A good estimation of *D' *is crucial for a better understanding of patterns of LD, a robust identification of haplotype blocks, more accurate algorithms for haplotype reconstruction, and better reproducibility of genetic studies. The popular MLE of *D' *is biased toward disequilibrium, and requires the use of thresholds on the MAF that have been shown to introduce ascertainment bias. By using an informative prior that models the LD between SNPs based on their physical distance, we define a Bayesian estimator that outperforms the MLE without increasing computational complexity. Our estimator is slightly biased toward equilibrium, but this bias tends to disappear quickly with increasing sample sizes, and at a faster rate than the bias toward disequilibrium of the MLE. Furthermore, our evaluation shows that the MAP estimator does not require any thresholds on the MAF.

There are several limitations to this work. The probability distribution of the haplotypes is modelled using a multinomial distribution with a Dirichlet prior, and this assumption can be relaxed to include more general models. Also, the prior distribution does not take into account recombination hotspots. We have assessed the impact of this assumption in our simulations, but more evaluation is needed. Our analysis is now limited to biallelic SNPs, however our Bayesian model can be extended to include measures of LD for multi-allelic SNPs. For example, a first-order approximation of the average estimator of *D' *suggested in [2] can be computed by averaging the MAP estimates. Some more work is needed to examine the effect of the prior hyper-parameters. In future work we will extend our results to other measures of LD, particularly *r*^2 ^= *D*/(*p*_*A*_*p*_*a*_*p*_*B*_*p*_*b*_). Some preliminary results that are posted in our supplementary web site suggest that a Bayesian estimator of *r*^2 ^developed along the line of the estimator introduced in this paper would gain robustness.

## Authors' contributions

PS developed the stochastic method, designed and carried out part of the simulations, and drafted the manuscript. MAG developed and evaluated the approximate method, and she implemented the method in a computer program. Both authors designed the study, and read and approved the final manuscript.
